# Clinical Characteristics of Patients Hospitalized with Coronavirus Disease, Thailand

**DOI:** 10.3201/eid2607.200598

**Published:** 2020-07

**Authors:** Wannarat A. Pongpirul, Joshua A. Mott, Joseph V. Woodring, Timothy M. Uyeki, John R. MacArthur, Apichart Vachiraphan, Pawita Suwanvattana, Sumonmal Uttayamakul, Supamit Chunsuttiwat, Tawee Chotpitayasunondh, Krit Pongpirul, Wisit Prasithsirikul

**Affiliations:** Bamrasnaradura Infectious Diseases Institute, Bangkok, Thailand (W.A. Pongpirul, A. Vachiraphan, P. Suwanvattana, S. Uttayamakul, W. Prasithsirikul);; US Centers for Disease Control and Prevention–Thailand Ministry of Public Health Collaboration, Bangkok (J.A. Mott, J.V. Woodring, J.R. MacArthur);; US Centers for Disease Control and Prevention, Atlanta, Georgia, USA (T.M. Uyeki);; Thailand Ministry of Public Health, Bangkok (S. Chunsuttiwat);; Queen Sirikit National Institute for Child Health, Bangkok (T. Chotpitayasunondh);; Chulalongkorn University, Bangkok (K. Pongpirul)

**Keywords:** reverse transcription PCR, viruses, severe acute respiratory syndrome coronavirus 2, SARS-CoV-2, SARS, COVID-19, 2019 novel coronavirus disease, zoonoses, viruses, coronavirus, Thailand, coronavirus disease

## Abstract

Among 11 patients in Thailand infected with severe acute respiratory syndrome coronavirus 2, we detected viral RNA in upper respiratory specimens a median of 14 days after illness onset and 9 days after fever resolution. We identified viral co-infections and an asymptomatic person with detectable virus RNA in serial tests. We describe implications for surveillance.

During January 2020, persons in Thailand were tested for the presence of severe acute respiratory syndrome coronavirus 2 (SARS-CoV-2) infection if they had a combination of fever or respiratory illness and a history of travel to Wuhan, China. Persons determined to be close contacts of a laboratory-confirmed coronavirus disease (COVID-19) case-patient also were tested during enrollment into contact tracing. Clinicians were able to request testing if they had a concern regarding persons who were exposed to travelers. During January 8–31, 2020, Bamrasnaradura Infectious Diseases Institute, the national infectious disease referral hospital in Bangkok, admitted 11 patients with laboratory-confirmed COVID-19. We describe clinical features, clinical management, and results of serial reverse transcription PCR (RT-PCR) testing for SARS-CoV-2 RNA for these patients.

## The Study

The 11 hospitalized patients had daily nasopharyngeal and oropharyngeal sampling for SARS-CoV-2 RNA testing. Specimens were collected by using synthetic fiber swabs, which were combined and placed into a single sterile tube containing ≈3 mL of viral transport medium. RNA was extracted and tested with conventional RT-PCR and real-time RT-PCR (rRT-PCR). We developed SARS-CoV-2–specific primers and probes by using a protocol from the World Health Organization (*1*) and validated results by using clinical specimens. Nasopharyngeal and oropharyngeal swabs and sputum specimens also were tested for 33 respiratory pathogens by using the Fast-Track Diagnostic rRT-PCR Respiratory Panel (Fast Track Diagnostics, http://www.fast-trackdiagnostics.com), according to the manufacturer’s instructions. During the study period, Thailand’s discharge criteria for hospitalized COVID-19 patients required resolution of clinical signs and symptoms and 2 respiratory specimens without detectable SARS-CoV-2 RNA collected >24 hours apart.

**Figure 1:**
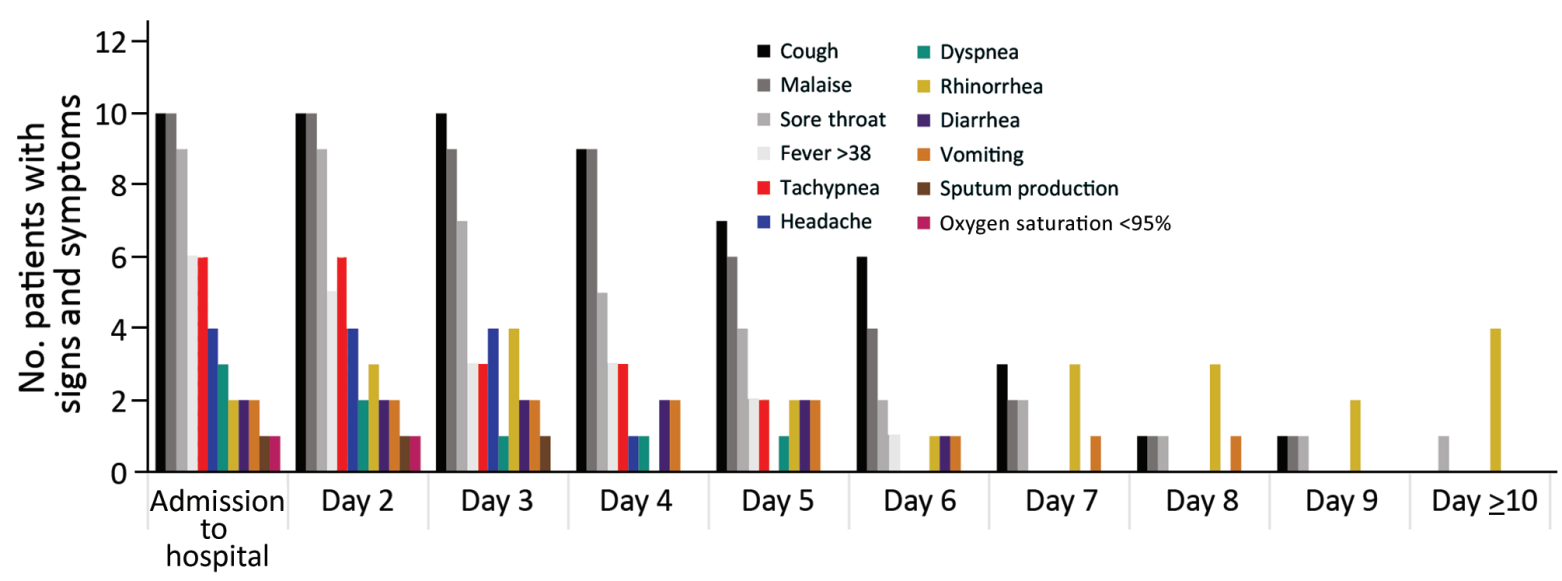
Number of patients with signs and symptoms by days following admission based on 11 patients with confirmed coronavirus disease, Bamrasnaradura Infectious Diseases Institute, Bangkok, Thailand, January 8–31, 2020

The median age of the patients was 61 years (range 28–74 years; Table 1). Cough, malaise, and sore throat were the most common signs and symptoms among the 11 patients (Figure 1). In patients with fever (temperature >38°C; 10/11), defervescence took a median of 6 days (4–11.5 days). Some patients had signs and symptoms that lasted >10 days (Figure 1; Table 2). Most patients received supportive care; none required mechanical or noninvasive ventilation during their hospitalization.

**Table 1:** Demographics, baseline characteristics, illness histories, laboratory values and treatment therapies of confirmed COVID-19 patients in Bamrasnaradura Infectious Diseases Institute, Bangkok, Thailand, 2020*

Demographics	Patient no.											Total, %	
	1	2	3	4	5	6	7	8	9	10†	11		
Age, y/sex	61/F	74/F	68/M	66/F	57/F	34/M	61/M	63/M	28/F	51/M	49/M	55 M/45 F	
Ethnicity	CH	CH	CH	CH	CH	CH	CH	CH	CH	TH	TH	82 CH/18 TH	
Occupation	Ret	Ret	Ret	Ret	Ret	EE	Ret	Ret	Tour guide	Taxi driver	Officer	54 Ret/46 other	
Detected through airport screening	Y	Y	Y	N	N	N	N	N	N	N	N	27 Y/73 N	
Detected through contact tracing	N	N	N	Y	N	N	N	N	N	N	N	9 Y/91 N	
Detected after patient voluntarily sought medical care	N	N	N	N	Y	Y	Y	Y	Y	Y	Y	64 Y/36 N	
Visited Hunan Seafood Market	N	N	N	N	N	N	N	N	N	N	N	0	
Underlying conditions													
Diabetes	N	N	N	N	N	Y	N	N	N	Y	N	18 Y/82 N	
Hypertension	Y	Y	N	Y	N	N	N	N	N	Y	N	36 Y/64 N	
COPD	N	N	N	N	N	N	N	N	N	N	N	0	
Asthma	N	N	N	N	N	N	N	N	N	N	N	0	
Cancer	N	N	N	N	N	N	N	N	N	N	N	0	
Cardiovascular disease	N	Y	N	Y	N	N	N	Y	N	N	N	27 Y/73 N	
Cerebrovascular disease	N	N	N	N	N	N	N	Y	N	N	N	9 Y/91 N	
Chronic liver disease	N	N	N	N	N	N	N	N	N	N	Y	9 Y/91 N	
Any chronic condition	Y	Y	N	Y	N	Y	N	Y	N	Y	Y	64 Y/36 N	
Current smoker	N	N	N	N	N	N	N	N	N	N	N	0	
Pregnant	NA	NA	NA	NA	NA	NA	NA	NA	N	NA	NA	0	
Laboratory values at time of admission (reference range)													
Leukocytes ×10^9^/L (4.5–8)	1.9↓	3.3↓	4	3.6	3.9	3.4↓	5.8	4.1	4.9	5.8	2.5↓		
Neutrophils, % (36–70)	48	64	66	63	56	80↑	63	83	73↑	58	54		
Lymphocytes, % (23–57)	40	19↓	25	25	33	1↓	30	16↓	23	31	30		
Platelets ×10^6^ /μL (140–400)	127↓	16.4↓	12.6↓	177	167	169	168	18.4↓	153	368	167		
Hemoglobin, g/dL (11–14)	13.3	12.8	11.5	13.1	13.2	13.3	15.3↑	13.8	11.4	14	14.8↑		
Hematocrit, % (35–41)	38	38	33↓	37	37.9	38	45↑	39	34	41	43↑		
ALT, U/L (0–31)	18	27	18	83↑	23	16	22	22	24	24	26		
AST, U/L (0–31)	14	12	15	47↑	16	19	20	14	25	16	22		
Other diagnostics													
Oxygen saturation on room air at admission	98	97	95	98	99	99	98	99	96	91↓	97		
Results from Biofire-33 multiplex PCR‡													
* Haemophilus influenzae*	+	+	–	–	+	+	–	–	–	–	–		
Adenovirus	–	*+*	–	–	–	–	–	–	–	–	–		
Influenza A	–	–	–	–	–	–	+	–	–	–	–		
* Klebsiella pneumoniae*	–	–	–	–	–	–	–	–	–	–	+		
Treatments													
Antimicrobial drugs, dose													
Ceftriaxone, 2 g 4×/d IV	1	0	7	0	0	0	7	0	0	7	0		
Ceftriaxone, 2 g/d orally	0	7	0	0	0	0	0	0	0	0	0		
AMOX/CLAV, 2 g 4×/d orally	6	0	0	0	0	0	0	0	0	0	7		
Oseltamivir, 150 mg 4×/d orally	5	0	0	0	0	0	5	0	0	0	5		
Nasal cannula, 5 L, no. days	0	0	0	0	0	0	0	0	0	3	0		
Duration of signs and symptoms reported at admission, d												Median (IQR)/ mean (SD)	
Cough	1	1	1	0	2	1	4	2	3	8	5	2 (1–4)/2.5 (2.3)	
Malaise or fatigue	4	2	4	0	2	4	13	2	3	5	5	4 (2–5)/4.0 (3.3)	
Fever	2	2	4	0	3	4	4	2	2	8	5	3 (2–4)/3.3 (2.1)	
Sore throat	4	0	3	0	3	2	4	2	3	7	5	3 (2–4)/3.0 (2.0)	
Rhinorrhea	2	2	4	0	2	1	3	2	2	2	4	2 (2–3)/2.2 (1.2)	
Headache	1	0	2	0	0	0	0	2	1	5	3	1 (0–2)/1.3 (1.6)	
Vomiting	0	1	1	0	0	0	0	1	0	0	0	0 (0–1)/0.3 (0.5)	
Diarrhea	0	0	1	0	0	0	0	1	0	0	0	0/0.2 (0.4)	

*ALT, alanine aminotransferase; AMOX/CLAV, amoxicillin/clavulanate; AST, aspartate aminotransferase; COVID-19, coronavirus disease; CH, Chinese; EE, electrical engineer; IV, intravenous; NA, not applicable; Ret, retired; TH, Thai; ↓, low; ↑, high; +, positive; –, negative.  †(*2*)  ‡BioFire Diagnostics (https://www.biofiredx.com)

**Table 2:** Clinical illness history and calculated intervals of confirmed COVID-19 patients in Bamrasnaradura Infectious Diseases Institute, Bangkok, Thailand, 2020*

Duration of signs and symptoms, d												Median no. days since symptom onset (IQR)	Mean no. days since symptom onset (SD)	T-test comparison between means
										

Patient no.										
1	2	3	4†	5	6	7	8	9	10	11
Onset of symptoms	0	0	0	NA	0	0	0	0	0	0	0	NA	NA	NA
Onset of fever	2	0	0	NA	0	0	9	0	1	0	0	0 (0–1.25)	1.2 (2.8)	–
First medical visit	4	2	4	0	3	4	13	2	3	3	2	3 (2–4)	3.6 (3.3)	–
Admitted to BIDI	4	2	4	0	3	4	13	2	3	8	5	4 (2–5)	4.4 (3.5)	–
SARS-CoV-2 RNA detected	4	2	4	0	3	4	13	2	3	8	5	4 (2–5)	4.4 (3.5)	–
Fever resolution	4	3	6	NA	6	4	13	4	6	13	11	6 (4–11.5)	7.0 (3.9)	–
Clinical resolution	13	10	11	NA	9	9	17	15	9	13	13	12 (9–13.5)	11.9 (2.8)	–
SARS-CoV-2 RNA undetectable	14	10	13	4	9	26	24	29	9	14	30	14 (9–26)	16.5 (9.1)	–
Discharge	15	11	14	6	15	29	26	32	11	16	33	15 (11–29)	18.9 (9.4)	–
Calculated intervals														
Admission to discharge	11	9	10	6	12	25	13	30	8	8	28	11 (8–25)	14.5 (8.7)	–
Fever resolution to SARS-COV-2 RNA undetectable	10	7	7	NA	3	22	11	25	3	1	19	9 (3–19.75)	10.8 (8.4)	–
Admission to fever resolution	0	1	2	NA	3	0	0	2	3	5	6	2 (0–3.5)	2.2 (2.1)	–
Fever resolution to clinical resolution	9	7	5	NA	3	5	4	11	3	0	2	5 (3–7.5)	4.9 (3.3)	–
Admission to clinical resolution	9	8	7	NA	6	5	4	13	6	5	8	7 (5–8.25)	7.1 (2.6)	–
Fever onset to fever resolution	2	3	6	NA	6	4	4	4	5	13	11	5 (4–7.25)	5.8 (3.5)	–
Admission to SARS-COV-2 RNA undetectable	10	8	9	4	6	22	11	27	6	6	25	10 (6–22.75)	12.2 (8.3)	–
Fever resolution to discharge	11	8	8	NA	9	25	13	28	5	3	22	10 (7–22.75)	13.2 (8.7)	–
Fever duration by stratified condition														
Patients detected through airport screening	2	3	6	–	–	–	–	–	–	–	–	3 (2–6)	3.7 (2.1)	0.14
Patients seeking medical care	–	–	–	–	6	4	4	4	5	13	11	5 (4–11)	6.7 (3.7)	–
Detectable SARS-COV-2 RNA duration by stratified condition														
Patients detected through airport screening	10	9	8	–	–	–	–	–	–	–	–	9 (8–10)	9.0 (1.0)	0.17
Patients seeking medical care	–	–	–	–	6	22	11	27	6	6	25	11 (6–25)	14.7 (9.6)	–

*COVID-19, coronavirus disease; NA, not applicable †Patient 4 was asymptomatic throughout hospitalization and PCR results reflects days with detectable SARS-CoV-2 RNA following admission.

**Figure 2:**
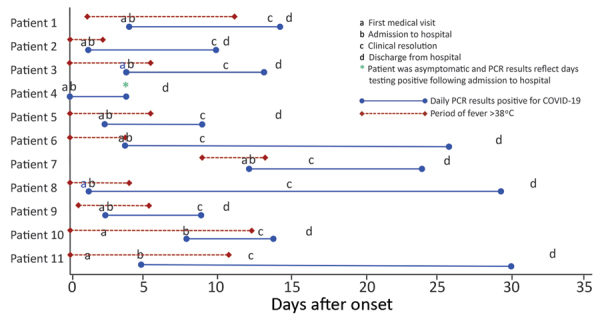
Clinical course for 11 patients with laboratory-confirmed COVID-19 by days since onset of their first symptom, Bamrasnaradura Infectious Diseases Institute, Bangkok, Thailand, January 2020. Blue solid bars indicate number of days each patient had detectable severe acute respiratory syndrome coronavirus 2 RNA. Red dashed bars indicate the number of days each patient had a fever >38°C. Asterisk denotes that patient 4 remained asymptomatic during hospitalization with detectable viral RNA for 4 consecutive days. COVID-19, coronavirus disease.

Patient 4 remained asymptomatic throughout hospitalization despite daily monitoring. However, her chest radiograph at admission revealed unilateral pneumonia (Appendix Figure). Patient 4’s nasopharyngeal and oropharyngeal specimens had detectable SARS-CoV-2 RNA on 4 consecutive days. She finally had 2 negative specimens separated by >24 hours and was discharged on day 7 after symptom onset (Figure 2). 

Patient 10, a taxi driver with no history of air travel, had the most severe clinical presentation among these cases (*2*). He reported close contact while transporting symptomatic travelers from China, a mechanism of exposure that has been described elsewhere (*3*–*5*). Patient 10 did not seek care for 10 days after his reported onset of fever. In Thailand, workers in the tourist industry, including those who transport tourists, are among the risk groups monitored for occupational exposures under updated clinical practice guidelines (*6*).

We detected viral co-infections in 2 patients during their hospitalization. Patient 2 had an adenovirus co-infection, and patient 7 had an influenza A virus co-infection (Table 1). Patient 7 was hospitalized for 13 days and influenza might have contributed to his clinical course. In Thailand, influenza A infection occurs most frequently during the rainy season, July–November (*7*). 

## Conclusions

We describe the clinical characteristics, clinical management, and laboratory findings from 11 COVID-19 patients hospitalized at Bamrasnaradura Infectious Diseases Institute. Most were febrile, but the onset of fever occurred early in the course of illness and fever resolution occurred 5 days before full clinical recovery and 10 days before discharge. Although no patient required mechanical ventilation or intubation, all had radiographic evidence of pneumonia, even those without respiratory symptoms. Together, these findings suggest that whereas fever and lower respiratory illness are commonly observed, case definitions requiring both fever and lower respiratory illness as signs and symptoms might not have detected several of these cases, especially later in the clinical course of illness.

Clinical resolution occurred a median of 12 (9–13.5) days after illness onset, and these patients had detectable SARS-CoV-2 RNA in upper respiratory tract specimens for a median of 14 (9–26) days after illness onset (Table 2). However, patients became afebrile 6 days after illness onset, with a median of 9 (3–19.75) additional days of detectable SARS-CoV-2 RNA in respiratory specimens after resolution of fever (Table 2). The required duration of hospitalization and observed period of viral RNA positivity for these patients underscore the potential burden of COVID-19 patients on hospital, diagnostic, treatment, and isolation capacities. Despite mild-to-moderate illness, the protracted period of SARS-CoV-2 RNA positivity in these patients’ specimens might indicate a lengthy period of infectiousness and highlights risks to providers caring for COVID-19 patients.

Among persons of Chinese ethnicity in our study, only 3/9 who traveled from China were detected through airport screening. During the study period, <7% of all persons under investigation for COVID-19 in Thailand were detected through airport screening (*8*). Given the proportion of cases identified through community surveillance, countries should not focus exclusively on point of entry screening or travel histories to detect cases of COVID-19, and maintaining healthcare providers’ awareness remains critical.

Patient 4 had detectable SARS-CoV-2 RNA for 4 consecutive days, but we were only able to follow her for 7 days before she returned to China. Her case is an example of a person without reported symptoms but radiologic evidence of disease and detectable virus over several days. Other studies have described asymptomatic patients with upper respiratory specimens positive for SARS-CoV-2 (*9*), and evidence suggests such cases pose a risk for transmission (*10**–**12*).

Our case series has some limitations. Patients could have recall bias regarding symptom onset before hospitalization. We were unable to complete a 14-day observation for some patients because they returned to China after discharge, including patient 4, who had no reported respiratory symptoms. 

The relatively long duration of hospitalizations in our study highlights the effects that current surveillance and isolation procedures can have on clinical care surge capacity. Duration of hospitalization was extended by Ministry of Public Health requirements for patients to remain in the hospital until symptom resolution and clearing of SARS-CoV-2 RNA in clinical samples. We observed that it took a median of 9 days to clear SARS-CoV-2 after fever resolution. In addition, we noted serial detection of SARS-CoV-2 RNA in respiratory specimens of an asymptomatic patient. 

Our observations of possible viral co-infections in COVID-19 patients and the resolution of fever relatively early during clinical course also have implications for surveillance strategies. Specifically, case definitions requiring fever could miss COVID-19 cases, especially later in the clinical course, and surveillance strategies that test only for SARS-CoV-2 could miss co-infections. Clinicians should consider the possibility of co-infection because the presence of other respiratory pathogens does not exclude the possibility of SARS-CoV-2 virus infection. Clinicians also need to better understand the relationship of RT-PCR detection of SARS-Cov-2 via multiple shedding routes (*13*) compared with the presence of culturable virus, especially in patients with few or no symptoms, because this might affect screening and isolation criteria. Whereas the current outbreak will undoubtedly change in character and magnitude, the information in this report could be combined with additional data sources to refine public health response and clinical management.

Appendix Additional information on hospitalized patients diagnosed with coronavirus disease, Thailand.
