# Supplementary material for: Clinical Characteristics of Patients Hospitalized with Coronavirus Disease, Thailand
Source: Emerg Infect Dis. 2020 Jul;26(7):1580–5. doi: 10.3201/eid2607.200598 (PMC7323520; doi:10.3201/eid2607.200598)
Supplement: Appendix — Additional information on hospitalized patients diagnosed with coronavirus disease, Thailand. [file 20-0598-Techapp-s1.pdf]

Article DOI: https://doi.org/10.3201/eid2607.200598

Clinical Characteristics of Hospitalized Patients Diagnosed with Coronavirus Disease, Thailand

Appendix

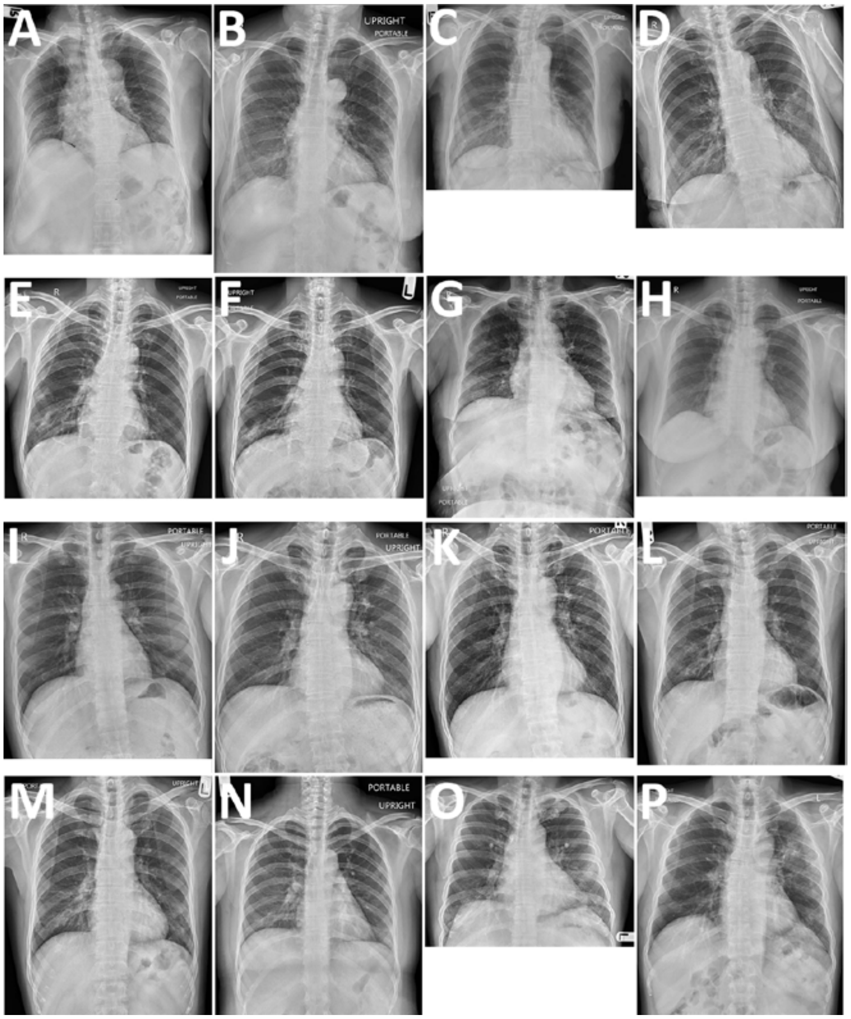

Appendix Figure. Chest radiographs of patients with confirmed severe acute respiratory syndrome coronavirus 2 infection, Bamrasnaradura Infectious Diseases Institute, Bangkok, Thailand, January–February 2020. A) Patient 1, a 61-year-old female at day 4 of symptom onset; radiologist interpretation: mild thickening lung marking at both lower lobes. B) Patient 1, at day 8 of symptom onset; radiologist interpretation: increased focal haziness at right upper lobe and left lower lobe. C) Patient 2, a 74-year-old

female at day 2 of symptom onset; radiologist interpretation: thickening of interstitial lung marking at both lower lobe fields. D) Patient 2, at day 6 of symptom onset; radiologist interpretation: recent reticular opacity at left middle lung field. E) Patient 3, a 68-year-old male at day 4 of symptom onset; radiologist interpretation: focal patchy opacity at right lower lobe. F) Patient 3, at day 9 of symptom onset; radiologist interpretation: no significant change. G) Patient 4, a 66-year-old female at the day of admission as she had no symptoms; radiologist interpretation: reticular opacity at right upper lobe. H) Patient 5, a 57-year-old female at day 3 of symptom onset; radiologist interpretation: increased lung marking at left lower lobe. I) Patient 6, a 34-year-old male at day 4 of symptom onset; radiologist interpretation: mild thickening lung marking at both perihilar region. J) Patient 7, a 61-year-old male at day 13 of symptom onset; radiologist interpretation: reticular opacity at left upper lung. K) Patient 7, at day 26 of symptom onset; radiologist interpretation: no significant change of reticular opacity at left upper lung. L) Patient 8, 63-year-old male at day 2 of symptom onset; radiologist interpretation: thickening lung marking in both lower lungs. M) Patient 8, at day 10 of symptom onset; radiologist interpretation: unchanged thickening lung marking in both lower lungs. N) Patient 9, a 28-year-old female at day 3 of symptom onset; radiologist interpretation: interstitial infiltration at right lower lung. O) Patient 10, a 51-year-old male at day 8 of symptom onset; radiologist interpretation: reticular opacity at left lower lung. P) Patient 11, a 49-year-old male at day 5 of symptom onset; radiologist interpretation: mild thickening lung marking at left lower lung.
